# Substance P – a regulatory peptide with defense and repair functions. Results and perspectives for the fight against COVID-19

**DOI:** 10.3389/fneur.2024.1370454

**Published:** 2024-05-30

**Authors:** Riffat Mehboob, Peter Oehme, Tehreem Anwar, Jens Peter von Kries

**Affiliations:** ^1^Lahore Medical Research Center, Lahore, Pakistan; ^2^National Heart Lung and Blood Institute, National Institute of Health, Bethesda, MD, United States; ^3^Retired, Berlin, Germany; ^4^Leibniz-Research Institute of Molecular Pharmacology (FMP), Berlin-Buch, Germany

**Keywords:** COVID-19, blood–brain-barrier, endothelial dysfunction, dysregulation immune response, Substance P, vasoactive peptides

## Abstract

Severe acute respiratory syndrome corona virus 2 (SARS CoV-2) is the cause of Corona virus disease 2019 (COVID-19), which turned into a pandemic in late 2019 and early 2020. SARS CoV-2 causes endothelial cell destruction and swelling, microthrombosis, constriction of capillaries, and malfunction of pericytes, all of which are detrimental to capillary integrity, angiogenesis, and healing processes. Cytokine storming has been connected to COVID-19 disease. Hypoxemia and tissue hypoxia may arise from impaired oxygen diffusion exchange in the lungs due to capillary damage and congestion. This personal view will look at how inflammation and capillary damage affect blood and tissue oxygenation, cognitive function, and the duration and intensity of COVID-19 disease. The general effects of microvascular injury, hypoxia, and capillary damage caused by COVID-19 in key organs are also covered in this point of view. Once initiated, this vicious cycle leads to diminished capillary function, which exacerbates inflammation and tissue damage, and increased inflammation due to hypoxia. Brain damage may result from low oxygen levels and high cytokines in brain tissue. In this paper we give a summary in this direction with focus on the role of the neuropeptide Substance P. On the basis of this, we discuss selected approaches to the question: “How Substance P is involved in the etiology of the COVID-19 and how results of our research could improve the prevention or therapy of corona? Thereby pointing out the role of Substance P in the post-corona syndrome and providing novel concepts for therapy and prevention.

## Introduction

Von Euler and Gaddum discovered Substance P (SP) in the year 1931 (1). They claimed that a hypotensive and spasmogenic component was present in equine brain and intestinal extracts. Later, it was discovered that the preparation, designated preparation P, was proteinaceous. Leeman’s team isolated SP from the hypothalamus of cattle and characterized it between 1970 and 1971 (2). SP performs a variety of physiological functions as a neuromodulator in addition to its responsibilities in inflammatory and immunological responses. Vascular dilation, smooth muscle contractions in the respiratory walls, and an increase in neural excitatory potential are all effects of it (3, 4). Bronchoconstriction may be brought on by SP in pathological situations (3). Notably, TAC-1, the gene that encodes SP, demonstrates unusual networking capabilities that make it prone to participation in a variety of illnesses, potentially fatal ones (5). Increased SP levels may be able to predict mortality and the severity of diseases including cancer, sudden infant death syndrome (SIDS), and traumatic brain injury (TBI) (6–9). In this publication we want to build a bridge between SP and the COVID-19.

### Substance P: a peptide with unusual features

It was a unique year for SP researchers when Nobel Symposium Stockholm was held in 1976 (10). One of the authors of this symposium was Peter Oehme. He theorized that the SP molecule encodes distinct information: one that acts directly on smooth muscle, sensory neurons, etc., and another that acts indirectly by influencing other transmitter systems, such as acetylcholine (11). The theory was verified by further study of Oehme’s group on the effects of SP on pain threshold, which revealed a dual effect (hyperalgesic in long response time and analgesic in short reaction time) (12, 13). Fascinating results were also obtained from the “SP-action on behavior” research that Oehme’s and Karl Hecht’s group conducted together. Rats were shown to respond normally to a range of stress models, including immobility, noise, electric footshocks, and others. These models included “decrease in learning,” “loss of deep sleep and REM sleep,” and “increase in blood pressure and heart rate” (14, 15). Therefore, Oehme and Hecht postulated an important role of SP as a regulatory peptide (regulide) in stress processes (14, 15). An interpretation of this “unusual features” was given by P. Oehme and W. A. Krivoy in (16). The Oehme group, together with Bruce Livett’s group, also looked at how SP interacted with the aminergic system. Both the nicotinic release of norepinephrine and the electrically induced release of acetylcholine were reduced by SP (17). In light of the literature’s knowledge that SP can cause peritoneal mast cells to release histamine and that SP is released from sensory nerves in response to antidromic stimulation, the Oehme group and the Pharmacological Institute of University College, London (UCL) started researching how to modify synaptic transmission in mast cells. This suggested that the release of histamine from mast cells required the whole SP molecule (18). On isolated peritoneal mast cells, the same structure–activity connections were seen (19). In 1987, Peter Oehme directed the efforts of his group in this direction and established a collaborative working group under the direction of Karen Nieber with the Research Institute of Lung Disease and Tuberculosis in Berlin-Buch. The well-known bronchospastic action of SP was of primary interest. In line with expectations, SP1–11 demonstrated a strong dose-dependent constrictor impact at the isolated guinea pig trachea’s basal tone (20). Consequently, a similar image to that of prior pharmacological research emerged. Oehme’s team thus intended to study the potential therapeutic or preventative benefits of N-terminal SP sequences and NK-1 receptor antagonists, with a focus on the respiratory tract. Furthermore, capsaicin’s impact on bronchial hyperreactivity made it interesting (21).

The active undecapeptide, SP, is first transformed enzymatically from a larger protein that is synthesized in the ribosome. In the central and peripheral neural systems of vertebrates, the peptide is broadly distributed. In the central nervous system, SP is hypothesized to play a role in controlling neuronal survival and ageing as well as a number of behavioral reactions (22). Since SP is the natural ligand with the highest affinity for the Neurokinin-1 Receptor (NK-1R), the biological effect of SP is primarily mediated through this receptor (23). The modulation of the vascular system, neuronal survival and degeneration, sensory perception, respiratory mechanism regulation, movement control, micturition, stomach motility, pain, inflammation, cancer, depression and salivation are the many functions that have been related to SP (24–30). It’s also important that SP operates independently on other cells in a paracrine and/or autocrine way, and that it may be found in bodily fluids such as blood, cerebrospinal fluid, breast milk, etc. SP mediates the communication between the immune and nervous systems. As a result, SP can control cellular activity through pathways that include autocrine, paracrine, endocrine, and/or neuroendocrine (23).

### SP-actions in the first defense line of the respiratory tract

Research results and data from Mehboob’s study support the concept that stem cell activity (SP) has a role in respiratory tract diseases such as COVID-19. These include infection and SP nociception symptoms, airway hypersensitivity/asthma in both phenomena, and varying patterns of COVID-19 disease severity in various age groups, which SP theory also addresses. Furthermore, the finding that viral load corresponds with SP secretion (31), explains the significant mortality rate among COVID-19 patients with diabetes, hypertension, and cardiac diseases. SP’s ventilatory function is well documented (32). SP was proposed by Riffat Mehboob as a possible component of the cytokine storming that happens after exposure to any foreign agent, such as the corona virus. Aprepitant, an NK-1R antagonist, has been suggested as a potential medicinal agent by inhibiting the receptor. As a result, it is speculated that SP may serve as a stimulant for cytokine storming during severe inflammation. Aprepitant is an FDA-approved medication for the treatment of chemotherapy-induced nausea and vomiting (33).

The most frequent cause of lower respiratory tract infections in infants, most prevalent virus responsible for bronchiolitis and an inflammation of the bronchioles is the respiratory syncytial virus (RSV). After intrapulmonary sensory nerve stimulation, RSV infection intensifies the inflammation (34). Additionally, NK-1R activity is increased in cases of RSV infection. The NK-1R can therefore be thought of as a key target for the therapy of the respiratory disorders because an increase of the SP/NK-1R system occurs in these diseases. The NK-1R and SP are both known to be elevated during inflammatory processes, and NK-1R antagonists have been shown to have anti-inflammatory effects in rats (23). SP is suggested to be an important mediator of neurogenic inflammation (35).

It has been noted that the number of SP-binding sites in the bronchial mucosa increases thrice and that SP/ NK-1R mRNA levels increase numerous times in RSV-infected lung (36). This impact may contribute to the inflammatory response to the virus and may be a target for the treatment of RSV disease and its potential complications, such as recurrent wheezing and pediatric asthma, utilizing NK-1 receptor antagonists (37). In the development of main and secondary immune responses to respiratory virus infections, lymphocyte NK-1R expression may be upregulated (38). Patients with sarcoidosis may produce more proinflammatory cytokines in their lungs, which would intensify localised pulmonary inflammatory responses if SP were to function through elevated NK-1R expression (39).

The airways contain considerable amounts of SP, which acts as a defense against inhaled irritants. The central nervous system reacts to unpleasant stimuli by causing a number of physiological changes, such as coughing, bronchoconstriction, hypotension, sleep apnea, and increased salivation. Additionally, prostaglandins, SP, and nitric oxide are released by the airway epithelium (40). Researchers have found higher amounts of NK-1R mRNA in broncho-alveolar lavage fluid, sputum samples, and lung tissue in diseases including asthma (41–43). For the airway hyperresponsiveness (AHR) to be mediated, SP and NK-1R must interact (44). Additionally, SP affects how the airways and lungs respond to ventilation, underscoring the extent of its effects on respiratory health (3, 33).

### SP and NK-1R’s function in immune response, inflammation and cytokine storm

Numerous cell types throughout the body, including vascular endothelial cells, fibroblasts, white blood cells, neurons, and regulatory organs for cardio-ventilation and respiration, express the seven-transmembrane domain receptor NK-1R. Inositol 1,4,5-trisphosphate (IP3) and diacylglycerol (DAG) are produced when SP binds to NK-1R, starting a signaling cascade. This chemical cascade paves the way for a complex web of immunological reactions (45, 46).

The function of SP and NK-1R in the activation of macrophages and other immune cells is particularly significant. The immunological response requires the activation of the NF-kB pathway and subsequent production of pro-inflammatory cytokines. This SP-mediated activation exemplifies the delicate interplay between the immunological and neurological systems by acting as a vital connection between them (47). Additionally, SP not only triggers immunological reactions but also feeds them by encouraging the release of other cytokines, resulting in a self-sustaining loop (48, 49).

SP has a significant impact on inflammation and interacts with the body in several ways. First of all, it promotes vasodilation and raises vascular permeability, making it simpler for immune cells to reach the damaged regions. Second, SP promotes leukocyte extravasation, facilitating the migration of immune cells to infection sites. Last but not least, SP directly affects both local and foreign cells, activating their immunological features (50).

Endothelial cells among many other cell types secrete SP, which is not just a function of certain immune cells (51). When SP is secreted, immune cells get activated and start producing cytokines, chemokines, and histamines, which are vital signaling molecules (52). The immune-suppressive cytokine TGF-1 is also inhibited by SP, which heightens the inflammatory response (53). Additionally, it increases the release of immunoglobulin by promoting the growth of T-lymphocytes, B-lymphocytes, and natural killer cells (54). Tumor necrosis factor-alpha (TNF-alpha) and interferon-gamma (IFN-gamma) can upregulate NK-1R in macrophages, enhancing the immune response (55).

Immune reactions are essential for defending the body against infections, but when they are out of control, they may be harmful. The “cytokine storm” phenomenon is a good example of this situation. The cytokine storm is a potentially fatal systemic inflammatory disorder and are characterized by high circulating cytokine levels and immune cell hyperactivation. Immune cells constantly release inflammatory mediators during a cytokine storm, which causes serious tissue damage and perhaps fatal situations. Since the cytokine storm is frequently linked to the acute respiratory distress syndrome (ARDS) experienced by infected individuals, the COVID-19 pandemic has drawn attention to it. The biggest danger in COVID-19 and other infections comes not from the virus but from an unchecked cytokine storm. It’s essential to stop or stop this storming effect to manage problems and enhance patient outcomes (34, 56).

**Figure 1:**
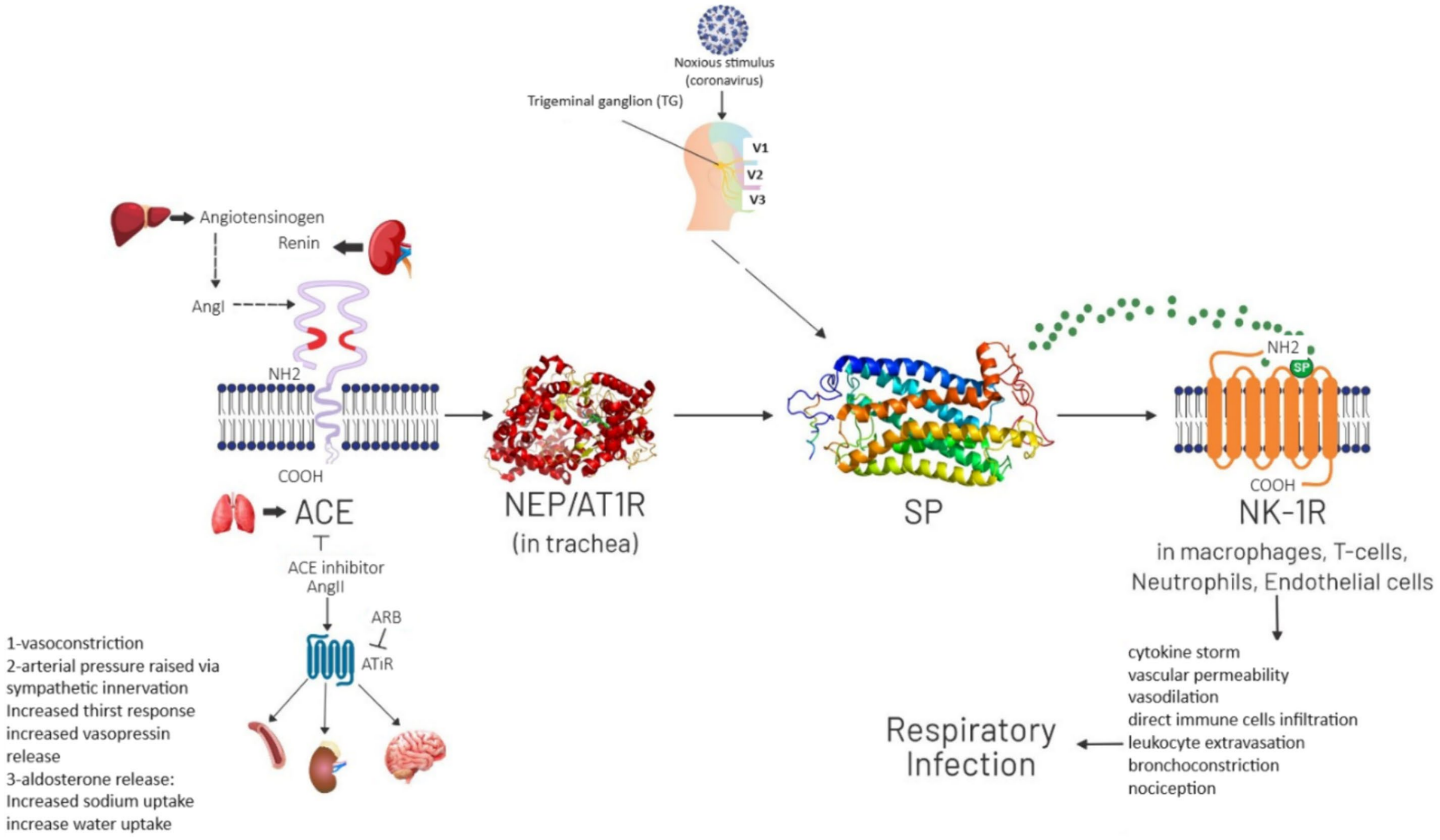
Pathway of SP involvement in respiratory infection.

The pathway through which SP acts in causing respiratory infection is shown in Figure 1. In normal physiological conditions SP is released from Trigeminal Ganglion. Neprilysin (NEP) degrades SP and it results in physiological processes like neuromodulation, neurotransmission and neurohormones. Noxious Stimulus such as COVID-19 attacks on angiotensin-converting enzyme (ACE) receptor that causes NEP to stop degrading SP. In result SP accumulation and binding with NK-1R causes pathological processes like cytokine storm, increased vascular permeability, vasodilation, direct immune cells infiltration, bronchoconstriction and nociception which then results in respiratory infection.

### Role of SP in neurological conditions

The involvement of SP as a potential mediator for long-term neurological consequences is also important in several scenarios. Parkinson’s disease (PD) and persistent post-COVID-19 olfactory impairment are two neurological illnesses associated with SP, a neuropeptide involved in neuroinflammatory processes. The research conducted by Schirinzi et al. investigated the presence of SP and its receptor NK-1R in olfactory neurons (ONs) of individuals with Parkinson’s disease (PD). This study examined the distinct correlation between gastrointestinal dysfunction in PD and the excessive production of SP. The significance of the SP/NK-1R pathway in PD is strengthened by its association with clinical markers, such as the Gastrointestinal Dysfunction Scale for PD and constipation. Additional investigation is required to verify the potential correlation between SP expression and intestinal inflammation associated with Parkinson’s disease. There was a suggestion that drugs licensed by the FDA may potentially modify SP as a target for therapeutic purposes (57). A study conducted by Schirinzi et al. unveiled significant new findings on the correlation between serum substance P (SP) levels and motor impairment in PD. A noteworthy finding was the association seen between the severity of motor impairments and elevated levels of SP in individuals with Parkinson’s disease. A notable discovery made during the discussion is the identification of SP as a potential biomarker or therapeutic agent for PD. It is critical to understand the study’s limitations, however, particularly the sample size and the absence of correlations with CSF biomarkers and other clinical features (58).

### Role of SP in COVID-19 and post-COVID complications

SP causes cytokine storming which is a primary cause of worsening of COVID-19. Immunomodulatory in nature, SP acts as a crucial channel between the immunological and neurological systems (48). All cytokines are produced by SP initially, and this further activates both SP and NK-1R (49). Three hypothesized pathways exist for SP to induce inflammation: (1) leukocyte extravasation; (2) vasodilation and vascular permeability; and (3) direct action on native cells and foreign invaders to activate their immunological characteristics (50). Immune system components including lymphocytes, neutrophils, dendritic cells endothelial cells and macrophages produce SP during inflammation (51). Mast cells emit histamines, chemokines, and cytokines as a consequence of SP activating immune cells (52). It induces inflammation by blocking the immune-suppressing cytokine TGF-β1, which is generated by macrophages (53). SP also have a role in olfactory neurons (ONs) and pathways that drive chronic post-COVID-19 olfactory dysfunction. SP is recognized to play a function in both starting and sustaining inflammatory responses. SP may be a crucial mediator in instances where chronic inflammation causes to long-term neurological consequences, such as post-COVID-19 difficulties and neurodegenerative illnesses like Parkinson’s disease. Schirinzi et al. explored a crucial and alarming outcome of continuous COVID-19: chronic olfactory impairment (OD). Overexpression of SP and Prokineticin-2 (PK2) in ONs of individuals with persistent post-COVID-19 olfactory impairment was identified, suggesting a key involvement. The relationship between PK2 levels and residual olfaction, as well as the theorized different functions of SP and PK2 in chronic inflammation and smell recovery (59).

### Role of endothelial cells

Within the pulmonary metabolism, lungs are essential for the conversion of several biochemical substances, including but not limited to adrenaline, angiotensin I and II, nitric oxide, bradykinin, prostaglandins, endothelin and others. When venous blood is changed into arterial blood, this transformation process takes place. The lungs function as an advanced filter that maintains the biochemical components of the dynamic hemodynamic system in a balanced and regulated manner (60). The endothelium of blood arteries functions as an endocrine tree in several organs, including the lungs. Targeted by coronavirus-2 are many important pathophysiological processes centered in one region. The main cellular target of viral aggression is the ACE-2 enzyme. Coronavirus inhibits ACE/ACE-2’s normal synthesis of angiotensin and bradykinins, which upsets the blood vessel’s equilibrium. It is necessary to comprehend the pathophysiology and molecular characteristics of COVID-19.

A COVID-19 disease and increased severity of respiratory distress are linked to endothelial dysfunction. Microthrombi and capillary hemorrhages inside the microcirculation are the first signs of vascular injury. In advanced stages of the illness, cytokine-induced endothelial dysfunction affects several organs and results in arterial hypertension, cardiac damage, diabetes, and neurological problems (61). It’s possible that NK-1R and SP contribute to the cytokine storming that cause’s endothelial dysfunction. Any painful stimulus to the body might cause an increase in SP levels in the circulation. This, in turn, causes an increased cytokine response, which leads to endothelial dysfunction. However, under normal physiological circumstances, the enzyme NEP indirectly contributes to endothelial dysfunction by breaking down SP (32, 62, 63).

Following an initial viral attack, the virus spreads to endothelial cells in the lungs and other organs in the setting of a COVID-19 disease. Endothelial dysfunction is most noticeable in the second or advanced stage of COVID-19 development. According to the STORM-2 hypothesis, there are biochemical pathways that have a deleterious influence on the endothelium of the lung, altering the coagulation system, vascular tone, hemodynamics, and arterial pressure control (64).

Aside from respiratory symptoms, the virus also has an impact on non-respiratory systems, most notably cardiovascular problems. According to previous studies, persons with severe COVID-19 disease often have underlying illnesses such as obesity, diabetes, cardiac issues, and hypertension. SARS-CoV-2 causes a cytokine storm, cellular damage, and a disruption in the renin-angiotensin system’s equilibrium, mainly in endothelial cells. COVID-19 disease is thus associated with endothelial dysfunction, thrombolytic and coagulation events, heart injury, hypoxia, and renal failure (65).

As described the very important cell target of the corona virus is the endothelial cell. Rudolf Virchow (1821–1902) the world-famous pathologist and founder of cellular pathology described the role of endothelial cells in the pathogenesis of disturbances in blood flow in a Trias. This was named “Virchow’sche Trias” (66). This means that three factors work together to interrupted blood flow: Hypercoagubility, stasis of blood flow, and endothelial injury. This trias is also important for the understanding of the COVID-19.

### The receptor for the coronavirus: ACE-2

**Figure 2:**
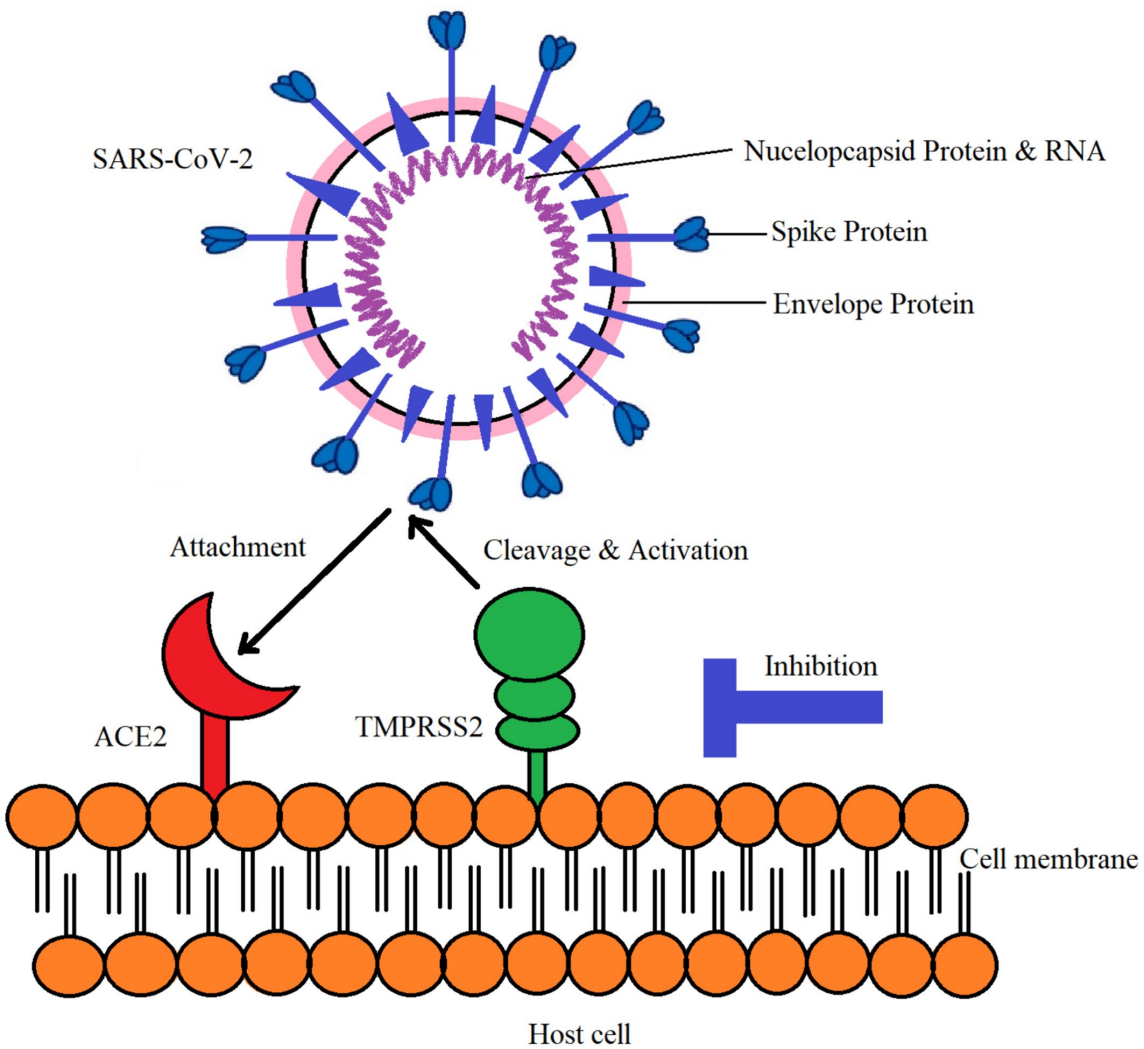
Interaction of SARS-CoV-2 with ACE-2 and TMPRSS2 in host cell.

ACE-2 was first discovered in 2000 and processes bradykinin, the major angiotensin polypeptide, and its different components (67, 68). Later research revealed that both ACE and ACE-2 are involved in the processing of these chemicals and have similar catalytic domains (Figure 2). However, ACE-2 is unable to hydrolyze neurotensin or bradykinin. ACE2 is receiving attention given that it is known to be a major factor in COVID-19 disease. Interestingly, SARS-CoV-2 may bind to ACE2, which is present in the host cell’s plasma membrane. Ten to twenty times higher binding affinity is possessed by SARS-CoV-2 compared to the initial strain of the virus (69). The SARS-CoV-2 coronavirus enters host cells via the ACE-2 receptor (70). While ACE-2 is damaged in many organs, SARS-CoV-2 is mostly identified in the lung’s alveolar epithelial cells (71).

SP is the first to respond to a hazardous stimuli and launches a swift defensive mechanism to preserve its life. It was shown that NK-1R deficient mice had decreased pulmonary inflammation when compared to controls (51). SP is secreted by immune cells and has actions that are autocrine, paracrine, and endocrine (72). It has the ability to stimulate distant cells, including fibroblasts, lymphatics, endothelial cells, smooth muscle cells, and white blood cells. It interacts with NK-1R and triggers the production of inflammatory mediators in the respiratory tracts by the endocrine and immune systems (73). Additionally, it is present on the phrenic nuclei and cardio-ventilatory regulatory centers, which control breathing and the diaphragm. It is mostly found in the brainstem nuclei that regulate breathing (46). The formation of the SP/NK-1R complex initiates a signaling cascade that yields DAG and IP3 (47).

NEP receptor research to cure COVID-19 was performed by Bellis et al. According to the research, COVID-19 induces ACE-2 down-regulation, which in turn results in a reduction in the breakdown of angiotensin II. This may produce an immediate lung and cardiovascular damage as well as a “cytokine storm.” Given that NEP is implicated in the breakdown of chemicals that prevent organ harm, they proposed that it could be a promising target for avoiding organ injury in COVID-19 patients (74). NEP contributes to the downregulation of SP, lowers inflammation, and bolsters Mehboob R’s hypothesis (32).

### Neuropilin-1: another viral entry point

Recently, it has been reported that Neuropilin-1 host receptor (NRP1) also serve as viral entry route (75). It’s a transmembrane glycoprotein, abundantly expressed in respiratory epithelium and its gene expression has been observed to be upregulated in the lung tissue of COVID-19 patients (76). Frthermore, its expression was raised in the olfactory epithelial cells of COVID-19 infected patients which may provide a viral entry passage toward the central nervous system (77).

### Use of NK1-antagonist against the cytokine storm As therapeutic and preventive strategy

Endothelial function and other associated issues may be improved by medications such as beta blockers, statins, and renin-angiotensin system (RAS) inhibitors. Furthermore, we propose a new therapeutic approach for the prevention and treatment of COVID-19 disease: the combination of an NK-1R inhibitor and the glucocorticoid dexamethosaone (62). Our prior study’s clinical trial produced really encouraging findings (78).

### NK-1R against cytokine storming

In a variety of medical circumstances, the use of NK1-antagonists to counteract the cytokine storm has emerged as a promising therapeutic approach. This strategy focuses on the SP and NK-1R complex, which is essential for controlling inflammation, immunological response, and other physiological functions. A G-protein-coupled receptor called NK-1R has a high affinity for the neuropeptide SP, which is present throughout the body. It is possible to harness the SP/NK-1R complex for therapeutic reasons by comprehending the mechanisms underlying how it affects immunological responses, inflammation, and other physiological processes.

A potential approach to the treatment of inflammatory disorders and certain viral infections involves the use of NK-1R-antagonists and capsaicin in the fight against the cytokine storm. NK-1R antagonists, which disrupt the communication pathway that leads to the production of pro-inflammatory cytokines, have shown their capacity to modify the immune response by acting on the neurokinin-1 receptor (79). This approach may lessen the intense inflammation seen during cytokine storms. These antagonists may decrease the production of cytokines like interleukin-6 (IL-6) and TNF-alpha, which are essential components of the cytokine storm’s damaging cascade, by blocking the NK-1R. Aprepitant and other NK-1 antagonists have demonstrated promising outcomes in clinical trials and preclinical studies when used as adjuvant therapies to decrease inflammation brought on by cytokine storms (80).

NK-1R antagonists, like aprepitant, Fosapitant, tardipitant have a lot of promise for treating cytokine storm disorders. Aprepitant, which is often used to treat nausea and vomiting brought on by chemotherapy, has come to light as a potential contender for controlling cytokine storms by inhibiting NK-1R. Aprepitant may lessen the production of pro-inflammatory cytokines including IL-6 and TNF-alpha, which are important mediators of cytokine storms, by blocking NK-1R signaling (80). Although further studies are required to completely prove its effectiveness in this situation, aprepitant’s immunomodulatory capabilities show promise as an additional treatment against inflammation brought on by cytokine storms.

On the other hand, dexamethasone, a strong corticosteroid, is a tried-and-true remedy for cytokine storms. Dexamethasone acts by lowering inflammatory responses of the immune system, which in turn lowers the synthesis of cytokines implicated in the cytokine storm cascade. In controlling cytokine storms connected to severe respiratory distress, such those seen in severe COVID-19 patients (81), it has been especially successful. Dexamethasone is regarded as a conventional therapy choice for illnesses characterized by cytokine storming since it has a strong clinical record of helping to reduce cytokine storms.

A research by Mehboob et al., evaluated a unique therapy method for severe to critical COVID-19 patients. The trial explored the combined use of aprepitant, an NK-1R antagonist, and dexamethasone, a corticosteroid, in controlling inflammation and enhancing respiratory recovery in COVID-19 individuals. The study revealed that the combination of aprepitant and dexamethasone has the potential to decrease inflammation by targeting the NK-1R and reducing the immune system’s inflammatory response. This combination medication was proposed as a new way to attenuate the cytokine storm, which is related with severe COVID-19 instances and respiratory distress (78). The research pointed out that SP, a neurotransmitter and neuromodulator, is produced from the trigeminal nerve in the brainstem in response to nociception (pain signaling) and has a direct role in respiratory disorders such as COVID-19. SP is linked in increased inflammation and the characteristic symptoms associated with the condition. The authors claimed that Aprepitant, when provided combined with the glucocorticosteroid dexamethasone, might help attenuate the inflammatory response by preventing NK-1R activation, hence possibly lowering the severity of COVID-19 (82).

### Neprilysin against cytokine storming

Given that NEP protects against pulmonary inflammatory responses and fibrosis, more research should focus on NEP’s possible involvement in the pathogenesis of COVID-19. There is less information on the use of NEP as a therapeutic agent since the majority of pre-clinical and clinical investigations in the medical profession focus on NEP inhibitors. The therapeutic and protective effects of NEP following lung damage are supported by earlier research. After the SARS-CoV-2 virus binds to the ACE-2 receptor on the surface of the cell, the lung may exhibit hyperplasia of pulmonary neuroendocrine cells together with the infiltration of many inflammatory cells. Excessive production of Gastrin-releasing peptide by the hyperplasia may promote the expression of the Gastrin-releasing peptide receptor on the surface of macrophages, leading to an increase in the release of inflammatory mediators that aid in the recruitment of neutrophils. NEP may block the release of inflammatory cytokines by degrading the gastrin-releasing peptide that is generated. NEP could be able to endure the strong cytokine storm. By stopping the breakdown of substance P, NEP inhibitors raise its levels. According to earlier post-mortem research, NEP activity was changed, which raised substance P’s half-life and elevated NEP expression in senile dementia (81). NEP has the ability to reduce the production of inflammatory cytokines, which may make target cells more susceptible to further SARS-CoV-2 viral activation. NEP may thereby increase tissue survival and improve lung histology (83, 84).

### ACE-2/AT1R against cytokine storm

Reduced levels of angiotensin- (1–7) and unopposed function of angiotensin II (AngII) might be the outcome of ACE2 internalization and the downregulation that follows (85). The SARS-CoV-2-mediated downregulation of ACE-2 and the ensuing elevated overall ratio of Ang II to angiotensin- (1–7) cause a decline in pulmonary function and lung injury because angiotensin- (1–7) plays a critical counter-regulatory role in many of the angiotensin type 1 receptor (AT1R)-related physiopathological functions (86, 87). Consequently, the renin-angiotensin-aldosterone system (RAAS) dysregulated angiotensin-II /AT1R axis and imbalanced ACE-2/ACE levels in COVID-19 may be partly to blame for the cytokine storm and subsequent pulmonary injury (88, 89). The effectiveness and safety of this medication have been studied in a few clinicopathological scenarios associated to ACE-2 decrement, including congestive heart failure (CHF) (90), ARDS (91, 92), and lung damage from viral illnesses such as RSV (93). The safety and effectiveness results that were published were encouraging. Human recombinant soluble ACE-2 (hrsACE-2) has been shown to be able to stop SARS-CoV-2 from entering human blood vessel and kidney organoids, according to a recent study by Monteil et al. (94) This discovery may point to a very promising therapeutic intervention to protect lung damage in COVID-19.

### Neuropilin receptor inhibitor

NRP1 inhibitor may provide a new therapeutic strategy to minimize SARS-CoV-2 infection (75). However, targeting NRP1 receptor alone would not be sufficient against COVID-19. Other receptors should also be targeted simultaneously for an effective treatment such as ACE-2 and NK1R inhibitors (95). We are of the view that vaccines may not be much effective due to the highly mutant nature of virus, instead, the use of broad spectrum and highly potent inhibitors against the host target receptors may be effective to curtain SARS-CoV-2. The purpose of these drug targets is to inhibit the entrance points for viruses and stop their vicious cascade of aggravating immune response and ultimate damage of host tissues.

### Perspectives for the future

The link between COVID-19 and SP seems to be an interesting field in future.

One noteworthy factor in the COVID-19 pandemic has been the considerable range in the severity of the illness across people. While age, comorbidities, and vaccination status are established variables increasing COVID-19 susceptibility, the function of neuropeptides like SP in regulating the immune response remains underexplored. SP, largely recognized for its function in neuroinflammatory processes and immunological modulation, may be a major component in determining an individual’s susceptibility to COVID-19. Recent investigations have revealed a possible link between low SP content in the blood and heightened sensitivity to COVID-19. This association might be attributable to SP’s involvement in controlling inflammation and modifying the immunological response. Researchers have observed that patients with lower SP levels may suffer a dysregulated immune response, resulting in increased viral replication and a more severe course of the illness.

This means more detailed studies to the relation of SP-concentration in blood and the lavage of the respiratory tract and the sensitivity against the coronaviren is important. Further research is also necessary to uncover possible biomarkers for COVID-19 sensitivity, allowing focused preventative interventions and therapies.

An interesting concept to further projects is the combination of the research to the COVID-19 with “stress research.” Facts to the role of Substance in stress responses exist a lot in the pioneer publications of P. Oehme and K. Hecht. Under chronic stress rats show lower SP-concentration in blood and different organs and a lot of disturbances in the cardiovascular functions and in the behavior (see first chapter of the publication and in the review (96). In relation to the COVID-19 is important, that SP can also normalize stress induced hyposomnia (97). One leading symptom in the post corona syndrome are disturbances in sleep. How is in such patients the SP level? What is with the effect of SP or partial sequences on hyposomnia in the post-corona syndrome?

Individuals with COVID-19 have several organ clinical symptoms as well as many post-COVID indications (98, 99). The endothelial dysfunction seen in patients with pre-existing comorbidities, such as obesity, diabetes, hypertension, or cardiovascular disease, seems to be a major factor in the etiology of COVID-19 (100, 101). Endothelial dysfunction, especially in individuals with co-morbidities such hypertension, diabetes, heart diseases, etc., may have a role in the etiology of COVID-19. To control AngII levels, ACE and its homolog, ACE-2, must be in equilibrium. Any alterations in the ACE/ACE-2 ratios and cytokine stress are associated with a malfunctioning endothelium system, which may result in vascular disorders.

For a better understanding of the effect of SP are investigations necessary: 1. to the action of SP and partial sequences on endothelial cells and 2.to the interaction of SP and Coronavirus on these cells and also on the angiogenesis. For such studies exist very good technical possibilities.

The Screening Unit (headed by Jens von Kries) at the Leibniz-Forschungsinstitut fuer Molekulare Pharmakologie established a leading open access technology platform for automated HTS-profiling of cell morphology alterations in response to cell function perturbations either by drug application or by RNA-interference or by CRISPR/Cas9 gene editing. The final aim of this is to extend the Cell Pathology concept of Rudolf Virchow by computer aided morphology pattern analysis and implication of AI. The platform already coordinates a network of European screening sites (EU-OPENSCREEN) for this purpose. One future focus in this is the morphology profiling of endothelial cells in response to COVID-19 infection and drug or gene function perturbation in combination. *In vitro* HUVEC cells form vessel like crosslinked network structures in Matrigel. After fluorescent staining of cell structures these can be analyzed via automated 2D or confocal 3D image capturing. This may introduce novel diagnostic and therapeutic tools against viral infection.

The comment made by Mehboob “Actually, the cytokine storming activated and initiated by SP is bringing about the disaster rather than the virus that is fatal and causing mortalities” (62) refers to past discussions on the appropriate control of epidemics by Rudolf Virchow, Robert Koch, Max von Pettenkofer, and Oscar Liebreich (102, 103). In light of the cholera outbreaks of the period, these talks might be summarized as follows: the disease germ, the vector, and the human interact and, hence, all three need to be taken into consideration equally. The germ alone is not the illness (104, 105). For a better therapy and prevention of the COVID-19 is the trias 1. Virus +2. Vector (air) + 3. Individual sensitivity the basis. The combination with the research to the regulatory peptide (regulide) Substance P with defense and also repair potencies could be very helpful.

## Data availability statement

The raw data supporting the conclusions of this article will be made available by the authors, without undue reservation.

## Author contributions

RM: Conceptualization, Project administration, Supervision, Writing – original draft, Writing – review & editing. PO: Conceptualization, Project administration, Supervision, Writing – original draft, Writing – review & editing. TA: Writing – original draft, Writing – review & editing. JK: Writing – original draft, Writing – review & editing.
